# The Environmental Consequences of Altered Nitrogen Cycling Resulting from Industrial Activity, Agricultural Production, and Population Growth in China

**DOI:** 10.1100/tsw.2001.400

**Published:** 2001-12-12

**Authors:** G.X. Xing, Z.L. Zhu

**Affiliations:** Institute of Soil Science, Chinese Academy of Sciences, Nanjing, China

**Keywords:** anthropogenic reactive N, nitrogen flux on land, environment, N_2_O, NO_3_

## Abstract

Human activities exerted very little effect on nitrogen (N) cycling in China before 1949. Between 1949 and 1999, however, rapid economic development and population growth led to dramatic changes in anthropogenic reactive N, inputted recycling N, N flux on land, N2O emission, and NH3 volatilization. Consequently, these changes have had a tremendous impact on the environment in China. In the current study, we estimated the amount of atmospheric wet N deposition and N transportation into water bodies from the watersheds and major valleys in China. Additionally, we addressed issues on leaching and accumulation of NO3- in the farmland under different climate zones, land use, and cropping systems as well as the potential influence of NO3- on underground water in China.

## INTRODUCTION

Since the 1950s, rapid industrial and agricultural development has occurred in China. As a result, China leads the world in output of major products (such as steel, coal, grain, and meat) and consumption of coal and synthetic fertilizers[1]. Meanwhile, the size of the population has increased from 0.54 billion in 1949 to 1.26 billion in 1999. This rapid economic development and expansion of the population have brought significant increases in anthropogenic reactive N and recycling N, and alterations of N cycling in watersheds[2,3]. The altered N cycling by human activities has led to increased N transportation into water bodies or escape into the atmosphere[2], which in turn has had significant environmental consequences. In particular, N pollution of water bodies[4] has caused euthrophication. Currently, most of the big lakes in China have stepped into the eutrophic stage[5]. The red tide has occurred more frequently and intensified year by year along the coast of China[6].

The adverse effects on the environment caused by altered N cycling due to increased human activities have already been observed in watersheds of China. China has a vast territory and diverse meteorological conditions ranging from the tropical zone in the south to the cold temperate zone in the north, and from the humid zone in the east to the arid zone in the northwest. Accordingly, crop plantation varies widely among different regions in China. Double- or triple-cropping systems are practiced in the east, the middle, and the south of China. Rice paddy fields account for one fourth of all the cultivated areas. Furthermore, although industrialization in China was initiated in the 1950s, about a century later than in the developed countries, the fast pace of development has far exceeded that of the developed countries. Accordingly, the increased human activities have exhibited a greater adverse impact on N cycling. N cycling and management, and their effects on the environment in the terms of natural and social factors, cropping systems, and the process of the economic development, have attracted tremendous attention from the scientific community.

## METHODS

### Calculation of Synthetic N and Fixation N by Legume Crops and Nonsymbiotic N in Paddy Fields

Legume crops in the study included soybean, peanut, pulse, and green manure. The consumption of synthetic N fertilizers, legume crop yields, and the cultivated area of green manure were cited from the *China Agriculture Yearbook*[7] while the essential data from 1949 to 1979 were available in the *Chinese Agriculture Yearbook* of 1980. The amount of fixation N by soybean, peanut, pulse bean, and green manure was calculated according to Zhu[8]. Nonsymbiotic N in paddy fields was calculated using the factor 45 kg N $ha^{-1}$ $year^{-1}$ as suggested by Zhu[8].

### Calculation of $NO_x$ Formed During Fossil Fuel Combustion

The total consumption of fossil fuel was referred to in the *Chinese Statistic Yearbook*, and the amount of $NO_x$ generated from the fossil fuel combustion was estimated using the conversion factors reported by Wang et al.[9]. Briefly, due to large varieties of fossil fuels and big differences in conversion factors for calculating $NO_x$ emission of the same type of fuels used in different industries, consumption of the fuels by different industries in China were worked out separately on a kind-by-kind basis. And then the $NO_x$ fluxes from combustion of coal, coke, crude petroleum, petrol, diesel oil, kerosene, residual oil, liquefied gas, and natural gas consumed in different industries were figured out and summed up separately according to three categories: coal, petroleum, and natural gas. Eventually, the $NO_x$ flux from the consumption of coal, petroleum, and natural gas between 1949 and 1999 was obtained individually.

### Calculation of Excrements N of the Domesticated Animal and Human Populations

The population of animals, such as cattle, horse, ass, mule, camel, swine, sheep, and poultry, and of humans was cited from the *Chinese Agriculture Yearbook* for the corresponding year. The conversion factors were used for calculation according to Xing and Yan[10]. Briefly, excrements N of swine, nondairy cattle, dairy cattle, sheep, poultry, and other domestic animals (horses, ass, mule, and camel) were calculated using the conversion factors 8, 42, 60, 7, 0.3, and 52 kg $N^{-1}$ $capital^{-1}$ $year^{-1}$, respectively. The excrements N of the human population were calculated by converting the whole human population into the adult population using the conversion factors 0.85 and 5 kg $N^{-1}$ $capital^{-1}$ $year^{-1}$[8].

### Calculation of $N_2O$ Emitted from Farmland in China

Direct $N_2O$ emission budget in farmland of China was calculated employing the factors and method suggested by IPCC Phase II methodology[11] and Xing and Yan[10]. Based on data observed at ten year-round monitoring posts scattered all over the country, extrapolation was also used for estimation[12].

### Calculation of $NH_3$ Volatilization in China

The method and conversion factors for the estimation of $NH_3$ volatilization in China were adopted according to Xing and Zhu[2] as specified in Table 1.

### Estimation of Atmospheric Wet N Deposition in China

The amount of atmospheric wet deposition in China was estimated based on mean annual precipitation from 1951 to 1990 and reported data[3,13,14,15]. In more detail, reports from 27 year-round monitoring posts scattered all over the country[13], 2 similar posts in the Shandong Province[14], and 1 in the Qinghai province in western China[15] indicated that the mean value of concentrations of $NO_3$ and $NH_4$ in precipitation was $4.36 \times 10^2$ mg N $m^{-3}$ and $1.35 \times 10^3$ mg N $m^{-3}$, respectively. The estimation was also based on the data of the mean annual precipitation from 1951 to 1990.

## RESULTS AND DISCUSSION

### Anthropogenic Reactive N Input in China the Past 5 Decades

Galloway et al.[16] proposed that the increased amount of symbiotic and nonsymbiotic N fixation due to expansion of cultivated areas for legume crops and rice was another source of anthropogenic reactive N. Since China is a major rice grower, we studied the four groups of anthropogenic reactive N includ-

**TABLE 1**
**$NH_3$ Volatilization Conversion Coefficient**

| Sources | | Emission Factors (kg N $kg^{-1}$ N) | References |
|---|---|---|---|
| Animal and human excretia N | | 0.20 | 11 |
| Synthetic fertilizer N | Upland: | 0.08 for urea and 0.10 for $NH_4HCO_3$ | 2 |
| | Paddy field: | 0.22 for urea and 0.28 for $NH_4HCO_3$ | |

ing synthetic N fertilizers entering the farm land, $NO_x$ formed during the fossil fuel combustion, symbiotic N fixed by legume crops, and nonsymbiotic N fixed in paddy fields. The variation in the amount of N grouped above was indicated in Table 2.

As shown in Table 2, the amount of anthropogenic reactive N in 1999 was 24 times larger than that in 1949. In the past 50 years, the increase of anthropogenic reactive N was mainly attributed to the large consumption of synthetic N fertilizers and $NO_x$ formed from fossil fuel combustion, especially the former. Consumption of synthetic N fertilizers increased 4000-fold while N mobilized by fossil fuel was 61 times greater than that in 1949. Of the total amount of anthropogenic reactive N in 1999, about 93% was from synthetic N fertilizers and $NO_x$ from the fossil fuel combustion. The situation, however, was the opposite in 1949. N from nonsymbiotic N in rice fields and N fixation by legume crops accounted for 93% while only 7% was from synthetic N fertilizers and $NO_x$ formed during fossil fuel combustion. It was concluded that industrial activities had very little effects on N cycling before 1949. The trend of a significant increase in anthropogenic reactive N started in the early 1980s when N mobilized by synthetic N fertilizers and fossil fuels made up of 85% of the total anthropogenic reactive N in China. These results demonstrate that industrialization is a dominant factor contributing to alterations in N cycling in China.

In contrast, nonsymbiotic N from rice fields decreased steadily from 1975 to 1999 whereas it had increased from 1949 to 1975. The decrease in nonsymbiotic N from rice fields after 1975 corresponds to reduced land areas for rice cultivation. Since the 1980s, the rapid expansion of population has created a dramatically increased demand for food and fibers accompanied by a significant yearly reduction of cultivated area of green manure. This has led to the reduced amount of symbiotic N fixed by legume crops. However, N fixed by legume crops has increased in a step manner since the 1990s mainly due to the expansion of areas for soybean, peanut, and other legume crops cultivation.

## Nitrogen Flux on Land in China the Past 50 Years

According to Howarth et al.[17], only two sources of N were considered to calculate N flux on land in China: the inputted synthetic N fertilizers and excrements N from domestic animals and from humans. Excrement N was a major contributor to recycling N. Like synthetic N fertilizers, it could enter water bodies directly. On the other hand, part of $NH_3$, $NO_x$, and $N_2O$ from excrements N could escape into atmosphere and deposit in other ecosystems. The N flux on land calculated on the basis of these two sources could reflect the degree of disturbance of human activities on N cycling and consequently the influence of N on the environment. In the present studies, we estimated the N flux on land in the past 50 years (Table 3) and its distribution in different regions in China.

As shown in Table 3, the N flux on land in China has increased from 586 to 4539 kg N $km^{-2}$ $year^{-1}$ steadily over the past 50 years. The excrements N of animals and of humans accounts for 99.89, 50.00, and 43.88% of the total N flux on land in China in 1949, 1985, and 1999, respectively. Therefore, it has had a significant impact on the environment such as the atmosphere and water bodies.

China has diverse climates and land usage, a high population density, and different levels of economic development among the different regions. In this study, the N flux on land was calculated region by region in accordance with these variables (Table 4).

**TABLE 2**
**Anthropogenic Reactive N in China from 1949 to 1999**

| Year | Synthetic N | N Fixed by Legume Crop | Nonsymbiotic N from Rice Fields | $NO_x$ from the Fossil Fuel Combustion | Total |
|---|---|---|---|---|---|
| | Tg N | | | | |
| 1949 | 0.006 | 0.55 | 0.69 | 0.08 | 1.33 |
| 1952 | 0.039 | 0.77 | 1.11 | 0.16 | 2.08 |
| 1957 | 0.32 | 0.91 | 1.28 | 0.33 | 2.84 |
| 1962 | 0.49 | 0.75 | 1.37 | 0.58 | 3.19 |
| 1965 | 1.33 | 1.15 | 1.49 | 0.65 | 4.62 |
| 1970 | 2.52 | 1.58 | 1.62 | 1.06 | 6.78 |
| 1975 | 3.40 | 1.65 | 1.95 | 1.68 | 8.68 |
| 1980 | 9.42 | 0.54 | 1.52 | 2.19 | 13.67 |
| 1985 | 12.68 | 0.68 | 1.44 | 2.94 | 17.74 |
| 1990 | 17.57 | 1.22 | 1.15 | 3.54 | 23.48 |
| 1995 | 22.22 | 1.87 | 1.12 | 4.29 | 29.5 |
| 1999 | 24.45 | 2.07 | — | 4.60 | 31.12 |

**TABLE 3**
**N Flux on Land in China from 1949 to 1999**

| Year | Synthetic N Fertilizers (Tg N) | Excrements N from Animals and Humans (Tg N) | N Flux on Land (kg N $km^{-2}$ $year^{-1}$) |
|---|---|---|---|
| 1949 | 0.006 | 5.62 | 586 |
| 1952 | 0.039 | 6.86 | 719 |
| 1957 | 0.32 | 8.17 | 884 |
| 1962 | 0.49 | 7.52 | 834 |
| 1965 | 1.33 | 9.00 | 1076 |
| 1970 | 2.52 | 10.15 | 1320 |
| 1975 | 3.40 | 11.33 | 1534 |
| 1980 | 9.42 | 12.18 | 2250 |
| 1985 | 12.68 | 12.96 | 1671 |
| 1990 | 17.57 | 14.88 | 3380 |
| 1995 | 22.22 | 17.91 | 4180 |
| 1999 | 24.45 | 19.12 | 4539 |

**TABLE 4**
**N Flux on Land in Different Regions in China (1995)**

| Country and Region | Area ($10^4$ $km^2$) | Population Density (individual $km^{-2}$) | Synthetic N (Tg N) | Excrements N (Tg N) | N Flux on Land (kg N $km^{-2}$ $year^{-1}$) |
|---|---|---|---|---|---|
| China | 960 | 123 | 22.22 | 17.91 | 4187 |
| Changjiang Valley | 181 | 226 | 7.30 | 5.32 | 8188 |
| Huanghe Valley | 75 | 101 | 1.77 | 1.44 | 4293 |
| Zhujiang Valley | 45 | 215 | 1.47 | 1.36 | 7244 |
| Eastern China[1] | 103 | 426 | 8.94 | 5.68 | 14,262 |
| Taihu Valley | 3 | 1080 | 0.72 | 0.18 | 30,000 |
| Central Western China[2] | 704 | 52 | 5.58 | 7.21 | 1816 |
| Remote Western China[3] | 406 | 10 | 0.77 | 2.53 | 622 |
| Tibet | 122 | 2 | 0.00089 | 0.39 | 327 |

[1] Includes provinces of Liaoning, Hebei, Shangdong, Jiangsu, Zhejing, Fujian, and Guangdong, and autonomous metropolitan cities of Beijing, Tianjin, and Shanghai.
[2] Includes provinces of Xining, Sichuang, Yunnan, Guizhou, Guangxi, Xinjiang, Qinghai, Gansu, Ningxia, Shanxi, Shannxi, and Mongolia.
[3] Includes autonomous regions of Tibet, Xinjiang, and Inner Mongolia.

As shown in Table 4 with respect to major river valleys, N flux on land of the Chanjiang Valley was higher than that of the Zhujiang Valley, which was higher than that of Huanhe Valley. N flux on land of the Chanjiang Valley and Zhujiang Valley was two times higher than the national average. Along the coast, the magnitude of N flux on land of the ten provinces (metropolises) reached 14,262 kg N $km^{-2}$ $year^{-1}$. The Taihu Valley had the highest N flux on land (30,000 kg N $km^{-2}$ $year^{-1}$). The inland provinces of western China has 1816 kg N $km^{-2}$ $year^{-1}$ of N flux on land, which was twofold lower than the national average. In the remote regions of western China, such as Tibet, Xinjiang, and Inner Mongolia, N flux on land was at 622 kg N $km^{-2}$ $year^{-1}$, which was eight times lower than the national average (Table 4).

N flux on land in China far exceeded that of other countries around the world. Howarth et al.[17] reported that the maximal N flux on land was found in the watersheds around the North Sea at 1450 kg N $km^{-2}$ $year^{-1}$. This value, however, was lower than the national average of China and even far lower than that of the provinces (metropolises) of eastern China along the coast (Table 4). Even the lowest N flux on land (327 kg N $km^{-2}$ $year^{-1}$) in China found in Tibet far exceeded the reported 76 kg N $km^{-2}$ $year^{-1}$ in Canada[17]. The markedly high N flux on land in China has already greatly influenced the environment of the coast and water bodies.

## Atmospheric N Deposition in China

Atmospheric deposition N, which derived from $NH_x$ and $NO_x$ in precipitation and dust has profoundly affected the terrestrial and oceanic ecosystems. In our current study, due to the lack of information on dry N deposition, only the wet atmospheric N deposition was calculated in Table 5. The amount of wet N deposition was estimated based on the data obtained in the early 1990s under the assumption that the current amount of wet N deposition was underestimated in Table 5 due to increases of anthropogenic reactive N input into watersheds and excrements N of animals and of humans in the late1990s (Table 2, Table 3).

The ratio of $NO_3$ to $NH_4$ of wet N deposition in China and its major river valleys was significantly higher than that of other countries in the world[18,19,20]. In China, the ratio of $NO_3$ to $NH_4$ was one third for both the Chanjiang and Zhujiang Valleys, one fourth for the Huanghe Valley, and one sixth for the Taihu Valley, demonstrating a high concentration of $NH_4$ in the precipitation. The large amount of wet N deposition in China could be attributable to the use of a specific type of synthetic N fertilizer made up of urea and ammonium bicarbonate. It accounts for 97.59% of the synthetic N fertilizer consumption, half of which is ammonium bicarbonate when volatized, giving rise to $NH_3$. High levels of $NH_3$ volatilization in China were also caused by the ill management of animal waste and excrements due to the lack of the well-equipped establishments. In the Huanghe Valley, the high ratio of $NO_3$ to $NH_4$ was a result of $NH_3$ volatilization of synthetic N fertilizers entering the farmland caused by calcareous soil with a high pH. In the Taihu Valley, the ratio of $NO_3$ to $NH_4$ was as low as one sixth. This might be related to the high N flux on land (30,000 kg N $km^{-2}$ $year^{-1}$) in this region (Table 4).

## $N_2O$ and $NH_3$ Emitted From Farmland in China the Past 50 Years

As an important greenhouse gas, 70 to 90% of $N_2O$ that escaped into the atmosphere originated from the soil[21]. The application of chemical N fertilizers was the major cause of $N_2O$ generation in agricultural land[12,22]. As a major agriculture producer, China stands first on the list in the consumption of synthetic N fertilizers.

In this study, direct $N_2O$ emission from agricultural fields and contributions of different N sources to $N_2O$ emission at different time points between 1949 and 1999 were estimated using the IPCC Phase II Methodology (1996) (Table 6).

As shown in Table 6, the direct $N_2O$ emission at 373 Gg $N_2O$-N from farmland in 1999 was 15 times higher than in 1949. The contribution of different N sources to $N_2O$ emission was also tabulated in Table 6. In 1999, animal waste and human excrements as fertilizers, synthetic N fertilizers, biological N fixation, and crop residue accounted for 13, 74, 7, and 6% of $N_2O$ emission respectively compared with 0.3, 51, 40, and 4% of each in 1949. Besides, the cultivated histosols accounted for 4% of $N_2O$ emission from farmland in China in 1949. In 1949, animal wastes, human excrements, and biological N fixation were the major sources of $N_2O$ emission, making up over 91% of the total. In contrast, synthetic N fertilizers had very little effect on $N_2O$ emission. However, the consumption of synthetic N fertilizers has increased sharply since 1980, and the contribution of synthetic N fertilizers to $N_2O$ emission from farmland rose to 67% of the total. In addition, the $N_2O$ emission from arable land and paddy fields in China was calculated based on data observed from agricultural fields (Table 7).

Table 7 demonstrated that 310 Gg N, which accounted for 78% of the total $N_2O$ emission, was emitted from arable land. The rest (88 Gg N, which accounted for 22%) originated from paddy fields, 35 Gg N (9% of the total emission) of which was emitted from paddy fields during the rice-growing season and 53 Gg N (accounting for 13% of the total) of which was emitted during the arable-land cropping season. The relatively high $N_2O$ emission from paddy fields in China was related to the

**TABLE 5**
**Atmospheric Wet Deposition N in Major Regions of China**

| Country and Region | Area | Precipitation | Wet N Deposition From $NH_4$ | Wet N Deposition From NOx | Wet N Deposition Total | |
|---|---|---|---|---|---|---|
| | ($10^4$ $km^2$) | ($10^3$ mm $year^{-1}$) | | (Tg N) | | $NO_3/NH_4$ |
| China | 960 | 0.63 | 8.28 | 2.68 | 11.36 | 1/3 |
| Changjiang Valley | 181 | 1.05 | 2.79 | 0.96 | 3.75 | 1/3 |
| Huanghe Valley | 75 | 0.49 | 0.52 | 0.12 | 0.64 | 1/4 |
| Zhujiang Valley | 45 | 1.44 | 0.96 | 0.33 | 1.29 | 1/3 |
| Taihu Valley | 3 | 1.1 | 0.0364 | 0.0062 | 0.043 | 1/6 |

**TABLE 6**
**Direct $N_2O$ Emission from Agricultural Fields in China Estimated by IPCC Phase II Methodology (1949 to 1999)**

| | Direct Soil Emissions (Gg N year$^{-1}$) | | | | | |
|---|---|---|---|---|---|---|
| Year | Synthetic Fertilizer | Animal Waste | Biological N Fixation | Group Residue | Cultivated Histosols | Total |
| 1949 | 0.07 | 13.4 | 10.4 | 1.1 | 0.95 | 26 |
| 1954 | 3.6 | 18.1 | 16.8 | 5.3 | 0.95 | 48 |
| 1960 | 5.5 | 17.6 | 15.3 | 4.8 | 0.95 | 44 |
| 1965 | 15.0 | 21.4 | 19.3 | 6.4 | 0.95 | 63 |
| 1970 | 28.2 | 24.6 | 28.5 | 7.5 | 0.95 | 90 |
| 1975 | 38.4 | 27.5 | 30.1 | 9.2 | 0.95 | 106 |
| 1980 | 106 | 28.5 | 12.5 | 11.2 | 0.95 | 159 |
| 1985 | 142 | 31.1 | 17.4 | 14.1 | 0.95 | 253 |
| 1990 | 196 | 35.1 | 17.5 | 16.9 | 0.95 | 266 |
| 1995 | 250 | 44.0 | 23.2 | 17.8 | 0.95 | 336 |
| 1999 | 275 | 47.8 | 25.8 | 23.2 | 0.95 | 373 |

**TABLE 7**
**$N_2O$ Emission from Cropland in China (Gg N year$^{-1}$)**

| Total | Arable land | | Paddy field | | $N_2O$ Emission during Rice Growing Season | | $N_2O$ Emission from Rice Field after Rice Harvest | |
|---|---|---|---|---|---|---|---|---|
| Emission | Emission | % Total | Emission | % Total | Emission | % Total from cropland | Emission | % Total from cropland |
| 398 | 310 | 78 | 88 | 22 | 35 | 9 | 54 | 13 |

Data from Xing[12].

paddy fields' unique systems of water management and cropping[12].

$NH_3$ volatilization is not only an important pathway of N loss in agriculture to the atmosphere in the form of $NH_3$, but it also affects the oxidation of $CH_4$, a key greenhouse gas in the atmosphere[23]. Furthermore, $NH_3$ is a major component of atmospheric dry/wet N deposition. Once deposited on terrestrial ecosystems, $NH_3$ functions as the second source of $N_2O$ despite of the enhanced nutrients on farmlands. Excessively deposited $NH_3$ in water ecosystems, however, will increase N load, which in turn will lead to adverse consequences. In the current study, variations of $NH_3$ volatilization in terrestrial parts of China over the past 50 years were estimated (Table 8).

As shown in Table 8, the amount of $NH_3$ volatilization has increased steadily in the past 50 years. Although the consumption of synthetic N fertilizers increased sharply year by year, the contribution of excrements of animals and of humans to terrestrial $NH_3$ volatilization was still greater than that of synthetic N fertilizers in China. In 1949, $NH_3$ volatilization derived from excrements N of animals and of humans accounted for almost 100% of the total $NH_3$ volatilized. Despite the significant increase in consumption of synthetic N fertilizers in 1999 (consumption has gone up to 24.44 Tg N) , only 40% of the total $NH_3$ volatilization originated from synthetic N fertilizers . About 60% of the total arose from animal and human excrements. Therefore, $NH_3$ volatilization generated from excrements N of animals and of humans has had significant effects on atmospheric environment, terrestrial ecosystems, and aquatic ecosystems.

## Transportation of N from Land to Water Bodies in China

Some observations on N transportation from land to water bodies were reported. Based on studies of the Changjang, Zhujiang, and Huanghe regions from 1980 to 1989, Duan et al.[24] calculated the amount of N transportation from these three rivers to the Pacific Ocean to be 0.78, 0.06, and 0.15 Tg, respectively. However, the estimation of Xing and Zhu[3] was 3.79 Tg for Changjiang, 0.84 Tg for Zhujiang, and 0.84 Tg for Huanghe.

**TABLE 8**
**$NH_3$ Volatilization in the Terrestrial Part of China over the Past 50 Years**

| Year | From Synthetic N Fertilizers | From Excrements N of Animals and Humans | Total |
|---|---|---|---|
| | (Tg) | | |
| 1949 | 0.0007 | 1.12 | 1.12 |
| 1952 | 0.0043 | 2.36 | 1.36 |
| 1957 | 0.004 | 2.63 | 1.63 |
| 1962 | 0.054 | 2.50 | 1.60 |
| 1965 | 0.146 | 2.80 | 1.95 |
| 1970 | 0.227 | 2.03 | 2.31 |
| 1975 | 0.374 | 2.27 | 2.64 |
| 1980 | 1.04 | 2.44 | 3.47 |
| 1985 | 1.39 | 2.59 | 3.99 |
| 1990 | 1.93 | 2.98 | 4.96 |
| 1995 | 1.44 | 3.58 | 6.04 |
| 1999 | 2.69 | 3.82 | 6.51 |

Considerable discrepancies in estimation of N transportation from land to water bodies calls for further investigation. Based on limited observation data, Zhu[25] recently estimated that the amount of N loss via farmland runoff and leaching were 1.24 and 0.5 Tg, respectively, which accounted for 5 and 2%, respectively, of applied synthetic N fertilizers in farmland.

N leaching in the farmland is a complex process. As discussed above, natural and social conditions differ greatly among different regions of China. Thus regions included in this study were categorized into three groups: paddy fields in southern China, the North China Plain, and the Loess Plateau to investigate N leaching and accumulation of N in the soil of farmland in China.

## *Nitrogen Leaching in Rice Paddy Fields of Southern China[26,27,28]*

Rice paddy fields cover one fourth of the total cultivated areas in China and are largely distributed in the south of the Changjiang River, which has an annual precipitation of 1000 to 2000 mm and an application rate of $ha^{-1}$ fertilizers at 400 to 600 kg N. In these areas, the paddy fields are cultivated under a paddy-arable land rotation system with the alternation of rice crops in the summer and other crops (such as wheat and oil rapes) in the winter.

Based on the data obtained, as shown in Table 9, the ratio of leaching $NO_3^-$ to the application rate of synthetic N fertilizers was not high regardless of the geographic locations of rice paddy fields (the north vs. the south of China).

It is clear that regardless of rice or wheat growing season, there was very little N leaching from applied N fertilizers in the farmland (ranging from 0.27 to 3.01%) in rice growing season. However, it was closely related to the amount of N fertilizers applied. Comparing two application rates showed that the larger the amount of N fertilizers applied, the higher the level of leaching N. The leaching N was slightly higher in wheat-growing season than in rice-growing season.

As reported by Lu et al.[29], estimation of leaching N derived from synthetic N fertilizers and endogenous soil N in rice paddy fields of southern China ranged from 5 to 10 kg N $ha^{-1}$ $year^{-1}$. This value is fairly low despite of the application of synthetic N fertilizers ranging between 400 and 500 kg N $year^{-1}$ $ha^{-1}$ to accommodate the double- or triple-cropping systems that dominate agricultural production in these areas. These results indicate that the level of leaching N derived from N fertilizers usage in rice paddy fields was low in southern China.

The low level of leaching N from synthetic N fertilizers in rice paddy fields could be attributable to two reasons: first, little $NO_3^-$ was generated during rice growing season due to the presence of flooding water above the ploughed soil layer; second, denitrification existed in the saturated underground soil layer of paddy fields in both rice and wheat growing season[4], suggesting that less $NO_3^-$ existed in the saturated underground soil layer.

In contrast, the amount of leaching N in the hilly arable land fields was much higher in southern China. Lu et al.[29] reported that the leaching N in the upland of red soil consisted of 15 to 27% of the total application of N fertilizers under an annual precipitation of 2182 mm. Since there is very limited information on N leaching in southern China, the amount of leaching in this region is not accurately known.

## *Leaching N in Arable Land of the North China Plain*

The North China Plain, located between 30 to 40° latitude N and 114 to 121° longitude E, is also known as the Huanghuaihai Plain. As one of the three major plains in China, the North China Plain

**TABLE 9**
**N Leaching in Rice Paddy Fields**

| Observation Sites | Application N (kg N ha$^{-1}$ year$^{-1}$) | % Leaching $NO_3^-$ to Application N: Rice Growing Season | % Leaching $NO_3^-$ to Application N: Wheat Growing Season | Observation Methods | Data Sources |
|---|---|---|---|---|---|
| Jiannin | 600[1] | 1.8 | 3.4 | Lysimeter | 26 |
| Changzhou | 254 | 0.56 | — | Lysimeter | 27 |
| | 388 | 3.01 | — | | |
| Shenyang | 150 | 0.27 | — | Lysimeter | 28 |
| | 250 | 0.46 | — | | |

[1] Indicates the application rate of N fertilizers during rice-wheat growing seasons.

covers an area of 0.3 million km$^2$ and is the alluvion formed by the Huang, Huai, and Hai Rivers. Most areas of this plain have semi-arid and semi-humid climates with an average annual rainfall of 640 mm. Two-crop planting once a year or three-crop planting every other year is a popular practice for this region, the biggest region for the cultivation of arable land crops. There have been very few results obtained on leaching N (Table 10).

Results summarized in Table 10 indicate that the level of leaching N in the arable land of the North China Plain was not high. Despite the better correlation of leaching N with the application rate of synthetic N fertilizers, great spatial variation still existed in the North China Plain. The ratio of leaching N to the applied N in farmland varied 2.0 to 17.2%[30,31,32]. The monsoon climate in the North China Plain could account for the low rate of leaching N. About 60 to 70% of rainfall could be accounted for in June, July, and August, the full growing stage of the crops. In this stage, crops consume much more $NO_3^-$ in soil than any other stages. Normally, crops planted in winter are subject to arid conditions during the growing period. The downward transport of $NO_3^-$ could only be observed in irrigation farmlands in the spring.

## *Leaching and Accumulation of $NO_3^-$ in Farmland of the Loess Plateau in China*

Between 34 to 40° latitude N and 102 to 114° longitude E locates the loess layer, which is 50 to 150 m in depth. With an average rainfall of 450 mm largely in July, August, and September, most areas are suitable only for single-crop planting once a year despite the fact that it is the biggest area for arable land crop production. Due to the little precipitation and the thickness of loess soil, the water table of underground water is 50 to 60 m lower than the surface soil, and in some areas, it can be 100 to 200 m lower. Although relatively much more $NO_3^-$ leaches from the ploughed layer into 20- to 60-cm-deep soil[33] or into 60- to 90-cm-deep soil[34], $NO_3^-$ in the surface soil of the Loess Plateau can only translocate downwards 1 to 1.5 m in depth during the fallow summer period[35].

Lu et al.[36] reported that the accumulation of $NO_3^-$ was within 0 to 4 m of the soil layer with different applications of N fertilizers and land usage. The accumulated $NO_3^-$ within 4 m reached 3414 kg N ha$^{-1}$ in a 8-year-old apple garden with the application rate of 900 kg N ha$^{-1}$ year$^{-1}$.

**TABLE 10**
**Leaching in Arable Land of the North China Plain**

| Observation Site | Application N (kg N ha$^{-1}$ year$^{-1}$) | Leaching N to N Applied (%) | Observation Methods | Data Sources |
|---|---|---|---|---|
| Lanfang | 173 | 3.6 | Lysimeter | 30 |
| Beijing | 150 | 0.9 | Lysimeter | 31 |
| | 225 | 2.0 | | |
| Beijing[1] | 75 | 1.8 | Lysimeter | 32 |
| | 300 | 17.2 | | |

[1] The averaged data from 1986 to 1992.

The accumulation of N in the soil layer under the ploughed layer was not only observed in farmlands of the Loess Plateau but also in arable land of the North China Plain. In the northern part of the Huanghuaihai Valley in the Beijing region, $NO_3$ accumulation happened mostly in the arable land soil above 0.4 m[37] with very little $NO_3$ moved into the soil layer under 1.4 m[38]. Chen et al.[39] reported that $NO_3$ in farmland could leach 1.3 m or deeper into the soil layer during corn growing season in the summer. In contrast, in wheat growing seasons, the accumulation of N in the soil layer fluctuated between 0.4 and 0.6 m in depth. In the present, the excessive exploitation of underground water has caused the water table to fall in this region. For instance, as reported by Wu[40], the table of underground water in the Beijing region has declined to 18 to 40 m at a rate of 1 to 2 m year$^{-1}$. It was hard for the downward $NO_3^-$ from ploughed soil to enter the underground water despite its potential endangerment to the underground water.

## N Pollution of Water Bodies in Developed Regions of China

N pollution of water bodies, one of the most serious environmental issues, has already raised alarming warnings in many regions of China, particularly the developed regions. As shown in Table 10, in the Taihu Valley, a developed region in the low reaches of the Changjiang River, N pollution of water bodies has deteriorated gradually.

As indicated in Table 11, N pollution in the river was worse than that of the lake. Inorganic N and $PO_4^{-3}$ exceeded their critical concentrations for eutrophication of lake water, particularly the N concentration. The shallow wells of 5 m or so for domestic usage in the rural area have an average concentration of 7.98 ± 9.65 mg $NO_3$-N l$^{-1}$. Of the wells under the investigation, 28% had $NO_3^-$ concentrations above the critical value of 10 mg $NO_3$-N l$^{-1}$. The potential sources of N pollution in the river and the lake as well as in wells of the Taihu Valley were reported by Xing et al.[4]. N pollutants in rivers and lakes were mainly from the domestic sewage and excrements of the domesticated animal and human populations while N pollutants in wells were from nitrogen leaching into farmlands[4]. However, the potential effect of dry/wet N deposition on the turnover of N in surface water (river and lake) could not be excluded. The amount of wet N deposition reached 0.043 Tg N km$^{-2}$ year$^{-1}$ (Table 5). The area of the Taihu Lake and Yangcheng Lake is over 2950 km$^2$. If calculated on the basis of the two lakes' maximum volume of 8 billion m$^3$, the wet N deposition could give a 0.46 mg l$^{-1}$ increase of N concentration every year.

## CONCLUSION

Human activities had trivial effects on N cycling in China 50 years ago,. The input of N in watersheds of China was mainly from the excrements of the animal and human populations, N fixation by legume crops, and nonsymbiotic N in farmland. Since the 1980s, however, the consumption of synthetic N fertilizers has increased drastically from 0.006 Tg N in 1949 to 9.42 Tg N in 1980, and again to 24.45 Tg N in 1999 whereas the excrements of animals and humans have only increased from 5.62 Tg N in 1949 to 19.12 Tg N in 1999. N flux on land ranged from 586 kg N km$^2$ year$^{-1}$ in 1949 to 4539 kg N km$^2$ year$^{-1}$ in 1999 in China, which was significantly higher than that of any other country. Great attention has been given to the N pollution of water bodies in China, the red tide in the estuary, and the eutrophication in lakes exacerbated year by year. Excessive inputs of N in water bodies might be responsible for it. The sources of nitrogen pollutants are mainly from the domestic sewage in cities and the discharge of the excrements of animals and humans into the water bodies. The sources could be also runoffs from farmland and atmospheric dry/wet N deposition.

N leaching in farmland varied widely among different regions due to the variations of climate, land use, and cropping systems. The amount of N leaching in farmland of the North China Plain and the northwestern Loess Plateau was not high. While it was even lower in the rice paddy field, it is relatively higher in the hilly upland in the humid climate zone of southern China. The movement of N from ploughed layer to underlayer soil was observed despite the limited depth of translocation.

The water table of underground water of the Loess Plateau is usually 50 m deep due to the extraordinarily thick loess soil, arid climate, and plateau topography. As a result of increased industrial and agricultural demand for water usage, excessive exploitation of underground water in North China Plain caused a more than 50-m reduction of the underground water table. Considering the continuous decline of the underground water table in the North China Plain and the Loess Plateau, the gradually accumulated N in the soil under the ploughed layer posed a

**TABLE 11**
**N Concentration in the River, Lake, and Well Water of the Taihu Valley (2000)**

| Water Body | $NO_3^-$ | $NH_4^+$ | Total N | $PO_4^{-3}$ |
|---|---|---|---|---|
| | (mg N l$^{-1}$) | | | (mg P l$^{-1}$) |
| River | 1.36 ± 0.68 (n = 23) | 5.69 ± 4.26 (n = 23) | 7.99 ± 4.44 (n = 23) | 0.20 ± 0.24 (n = 13) |
| Lake[1] | 1.27 ± 0.98 (n = 7) | 0.33 ± 0.43 (n = 7) | 1.88 ± 1.46 (n = 7) | 0.022 ± 0.085 (n = 7) |
| Well | 7.89 ± 9.65 (n = 40) | <0.2 | | |

[1] The Taihu Lake and the Yangcheng Lake.

potential threaten to the underground water even though it is unlikely that $NO_3^-$ may leach into the underground water bodies within a short period of time. Although there are some reports on $NO_3^-$ pollution of underground water in some regions of North China, nitrogen pollutants affect more the surface water than the underground water.

Of the total rice paddy fields in China, about 86% are cultivated under a paddy-arable land rotation system. During the rice-growing period, at least one cycle of dry-and-wet alternation is practiced in rice paddy fields. The unique cropping systems have made paddy fields an important source of $N_2O$, which consists of 22% of the total N emission from agricultural fields in China. Great uncertainties yet reside in what and how atmospheric dry/wet N deposition affects the terrestrial ecosystems such as forest, grassland, and natural wetlands, and further studies should be carried out in the near future.

## ACKNOWLEDGEMENTS

This work was supported by the grants (49271042, 39790110, and 49971048) from National Natural Science Foundation of China.

| | | |
|---|---|---|
| Received: | July | 21, 2001 |
| Revised: | November | 14, 2001 |
| Accepted: | November | 14, 2001 |
| Published: | December | 12, 2001 |
